# A204 DUPILUMAB IMPROVES HISTOLOGIC, ENDOSCOPIC, AND SYMPTOMATIC ASPECTS OF EOSINOPHILIC ESOPHAGITIS IN PATIENTS AGES 1–ampersand:003C12 YEARS

**DOI:** 10.1093/jcag/gwad061.204

**Published:** 2024-02-14

**Authors:** M Chehade, E Dellon, J M Spergel, M E Rothenberg, R D Pesek, M H Collins, I Hirano, R Liu, E Laws, E Mortensen, R Abdulai, J Maloney, E McCann, M P Kosloski, J D Hamilton, C Samuely, L Glotfelty, A Shabbir, M Chow

**Affiliations:** Icahn School of Medicine at Mount Sinai, New York, NY; University of North Carolina School of Medicine, Chapel Hill, NC; Children’s Hospital of Philadelphia, Philadelphia, PA; Cincinnati Children’s Hospital Medical Center/University of Cincinnati College of Medicine, Cincinnati, OH; University of Arkansas for Medical Sciences/Arkansas Children's Hospital, Little Rock, AR; Cincinnati Children’s Hospital Medical Center/University of Cincinnati College of Medicine, Cincinnati, OH; Northwestern University Feinberg School of Medicine, Chicago, IL; Regeneron Pharmaceuticals Inc., Tarrytown, NY; Sanofi, Bridgewater, NJ; Regeneron Pharmaceuticals Inc., Tarrytown, NY; Sanofi, Bridgewater, NJ; Regeneron Pharmaceuticals Inc., Tarrytown, NY; Regeneron Pharmaceuticals Inc., Tarrytown, NY; Regeneron Pharmaceuticals Inc., Tarrytown, NY; Regeneron Pharmaceuticals Inc., Tarrytown, NY; Regeneron Pharmaceuticals Inc., Tarrytown, NY; Sanofi, Bridgewater, NJ; Regeneron Pharmaceuticals Inc., Tarrytown, NY; Sanofi, Mississauga, ON, Canada

## Abstract

**Background:**

Dupilumab is approved in Canada for eosinophilic esophagitis (EoE) in patients (pts) aged ≥12 years who weigh ≥40 kg; no treatments are approved for pts aged ampersand:003C12 years.

**Aims:**

The Phase 3 EoE KIDS trial evaluated dupilumab efficacy and safety vs placebo (PBO) in pts aged 1–ampersand:003C12 years with EoE.

**Methods:**

Part A was a 16-week (W) PBO-controlled study; pts were randomized 2:2:1:1 to weight-tiered dupilumab on a higher-exposure (HE) or lower-exposure (LE) regimen, or PBO (2 groups) to W16. In Part B, pts continued dupilumab, or switched from PBO to dupilumab (HE/LE), to W52.

**Results:**

At W16 (Part A), the HE dupilumab group had an improved proportion of pts achieving peak esophageal intraepithelial eosinophil count (PEC) ≤6 eosinophils/high-power field (hpf) vs PBO (67.6% vs 2.9%, *P*ampersand:003C0.0001; primary endpoint). At W52 (Part B), 62.9% of HE dupilumab/dupilumab pts, and 52.9% of PBO/HE dupilumab pts, achieved PEC ≤6. At W16, the following improved with HE dupilumab vs PBO: EoE-Histologic Scoring System grade and stage scores (–0.88 vs +0.02 and –0.84 vs +0.05, respectively, both *P*ampersand:003C0.0001); EoE-Endoscopic Reference Score (–3.5 vs +0.3, *P*ampersand:003C0.0001); overall caregiver assessed EoE status (global impression of change mean [SD], 2.1 [1.2] vs 3.3 [1.4]; lower scores represent more improvement). Improvements were maintained with continuous dupilumab and improved in PBO/dupilumab pts at W52. LE dupilumab maintained similar improvements achieved through W52; however, effects were generally either comparable or numerically lower with LE dupilumab vs HE dupilumab. At W16, COVID-19, rash, headache, and injection-site erythema occurred more frequently in patients receiving dupilumab, with higher incidence than PBO; AEs were similar through W52.

**Conclusions:**

HE dupilumab met the primary endpoint of PEC ≤6 eos/hpf vs PBO, demonstrated significant changes in histologic and endoscopic aspects of EoE, and improved symptoms with benefits maintained/increased to W52.

**Funding Agencies:**

Research sponsored by Sanofi and Regeneron Pharmaceuticals Inc.

